# Implantable Membrane Sensors and Long-Range Wireless Electronics for Continuous Monitoring of Stent Edge Restenosis

**DOI:** 10.1021/acsami.5c08212

**Published:** 2025-07-03

**Authors:** Allison Bateman, Yuheng He, Chris Cherono, Jimin Lee, Nima Ghalichechian, Woon-Hong Yeo

**Affiliations:** † George W. Woodruff School of Mechanical Engineering, 115724 Georgia Institute of Technology, Atlanta, GA 30332, United States; ‡ Wearable Intelligent Systems and Healthcare Center (WISH Center) at the Institute for Matter and Systems, Georgia Institute of Technology, Atlanta, GA 30332, United States; § School of Electrical and Computer Engineering, 1372 Georgia Institute of Technology, Atlanta, GA 30332, United States; ∥ Wallace H. Coulter Department of Biomedical Engineering, Georgia Institute of Technology and Emory University School of Medicine, Atlanta, GA 30332, United States; ⊥ Parker H. Petit Institute for Bioengineering and Biosciences, Georgia Institute of Technology, Atlanta, GA 30332, United States; # Korea KIAT-Georgia Tech Semiconductor Electronics Center at the Institute for Matter and Systems, Georgia Institute of Technology, Atlanta, GA 30332, United States

**Keywords:** vascular stent monitoring, implantable bioelectronic sensor, wireless restenosis detection, monostatic reading, conformable flexible pressure sensor

## Abstract

Continuous monitoring of vascular stent health is crucial for high-risk patients. Current approaches predominantly rely on inductive coupling, which limits the wireless reading distance and requires precise antenna alignment with implanted stents. Here, we present a long-range wireless electronic system that incorporates a low-resistance inductive stent fabricated via laser micromachining and electroplating of biocompatible metal films, forming a mechanically robust and conductive interface optimized for radar interrogation. A conformal, flexible capacitive pressure sensor is developed from soft dielectric elastomers and integrated within the stent’s structure, enabling localized detection of hemodynamic pressure changes indicative of stent-edge restenosis. Computational modeling validates the antenna design as an effective radiator, while an in vitro study evaluates the performance of the stent-sensor assembly and wireless coupling. Our system successfully detected the stent from a distance of 50 cm, providing trackable localized pressure signals at 2 GHz for healthy stents as well as diagnostic capabilities for 50% stent-edge restenosis. This work establishes a new class of enhanced wireless stents, offering extended readout distances and real-time diagnostic capability, with broad implications for developing next-generation bioelectronic interfaces.

## Graphical abstract

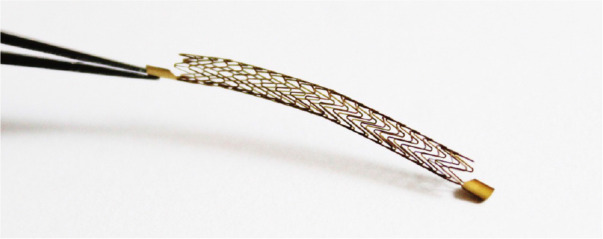

## Introduction

Cardiovascular disease is the leading cause of death worldwide, accounting for an estimated 20.5 million deaths in 2021.^1^ The most common form, atherosclerosis, occurs when arteries narrow due to plaque buildup, increasing the risk of heart attacks and strokes. A standard treatment for atherosclerosis is angioplasty in which a metal mesh stent is inserted into the narrowed artery to restore blood flow. In the United States alone, over 600,000 patients undergo this procedure annually.^2^ However, while stents effectively address initial blockages, patients remain at risk of further narrowing, either within the stent or along the treated artery, due to obstructive tissue growth. With drug-eluting stents (DES) replacing bare-metal stents, the probability of developing in-stent restenosis has dramatically decreased.^3^ Unfortunately, the implantation of DES does not fully prevent tissue regrowth at the edge of the stents, known as stent edge restenosis (SER).^4^ This condition often arises from the balloon angioplasty procedure, where the inflation of the balloon causes local damage to the edge of the stent boundaries, increasing the inflammatory response and local tissue buildup.^5^ Currently, the only means to monitor stent health is through in-hospital procedures such as intravascular ultrasound, optical coherence tomography, or echocardiography.^6^ These methods are not only costly and resource-intensive but also often detect issues too late, as patients may remain asymptomatic until complications become severe and life-threatening.

Previous studies have been conducted to monitor stent health through wireless means.^7–13^ Studies have shown success in using near-field inductive coupling as a method; however, the reading distance limits the feasibility of this approach for in vivo applications. Furthermore, many studies that optimize antenna capabilities have not extensively examined stents in an in vitro environment, thereby restricting their applicability for disease diagnosis.^14–16^ Other research has highlighted implantable antennas that incorporate energy harvesting modules.^17^ This reading method shows promising applications in digestible systems; however, the size and complexity of the required circuitry pose challenges for vascular applications. These limitations emphasize the need for a wireless system capable of reliably detecting SER within a patient while addressing challenges related to size, feasibility, and in vivo applicability.

Here, we present implantable membrane sensors and long-range wireless electronics for continuous monitoring of SER. This work demonstrates a stent capable of being detected 50 cm away from a receiving antenna, with the ability to diagnose stent edge restenosis of 50% by monitoring local pressure changes. The full-wave electromagnetic simulation studies validate the proposed reading scheme, along with preliminary studies using a balloon catheter to pressurize the stent. Finally, we conducted the testing using a pulsatile flow machine to mimic pressure changes per heartbeat and show continuous detection of the stent and SER diagnostic capabilities.

## Results and Discussion

### Material Preparation and Device Fabrication

The device is designed as an LC circuit to wirelessly communicate with the receiving antenna and diagnose for SER (Figure 1a). As seen in Figure 1b,c, the stent is designed to function as an inductor by removing all of the connecting bridges. Removing these bridges allows the stent to enable current flow in a 3D helical pattern and act as an inductor for the LC circuit. This stent is designed to be the same size as an average coronary stent, with a length of 27 mm and an expanded diameter of 4 mm. This inductive strut design has been reported in previous works.^9,12,18^ To fabricate the stent, it is first laser-cut with a femtosecond laser and then postprocessed to minimize resistance, enabling wireless data to be read from a greater distance (Figure 1d and Video S1). The postprocessing starts with sonication and electropolishing, which cleans the laser-cutting abrasions and results in a uniform electroplated film (Figure S1). The stent is initially electroplated with copper, which provides a conductive adhesion layer before gold electroplating. It also lowers the resistance and prepares the stent for the final thin coat of gold, which improves biocompatibility and reduces the resistance to below 2 Ω (Figures S2 and S3). This process allows the resistance to be lowered by almost 10 times (Figure S4). The stent is finally coated in parylene-C to insulate the conductive films and provide an additional layer of biocompatibility. The stent was imaged using field emission scanning electron microscopy (FE-SEM) (Figure 1e). Energy dispersive spectroscopy (EDS) was used to evaluate the elemental composition of the varying layers of the device. As seen in Figure 1f, the copper layer coats the stainless-steel base, which is shown here with a high concentration of iron, as expected.^19^ Copper and gold layers are approximately 30 μm and 10 μm thick, respectively. The parylene-C (C_16_H_14_Cl_2_), which is easily distinguishable by the presence of chlorine (Cl), coats the gold layer of the stent.^20^ Additional data are presented in Figures S5 and S6. The materials exposed to the patient’s blood flow and tissues in this system have been previously studied for biocompatibility and did not display any cytotoxic response.^12,18^

**Figure 1:**
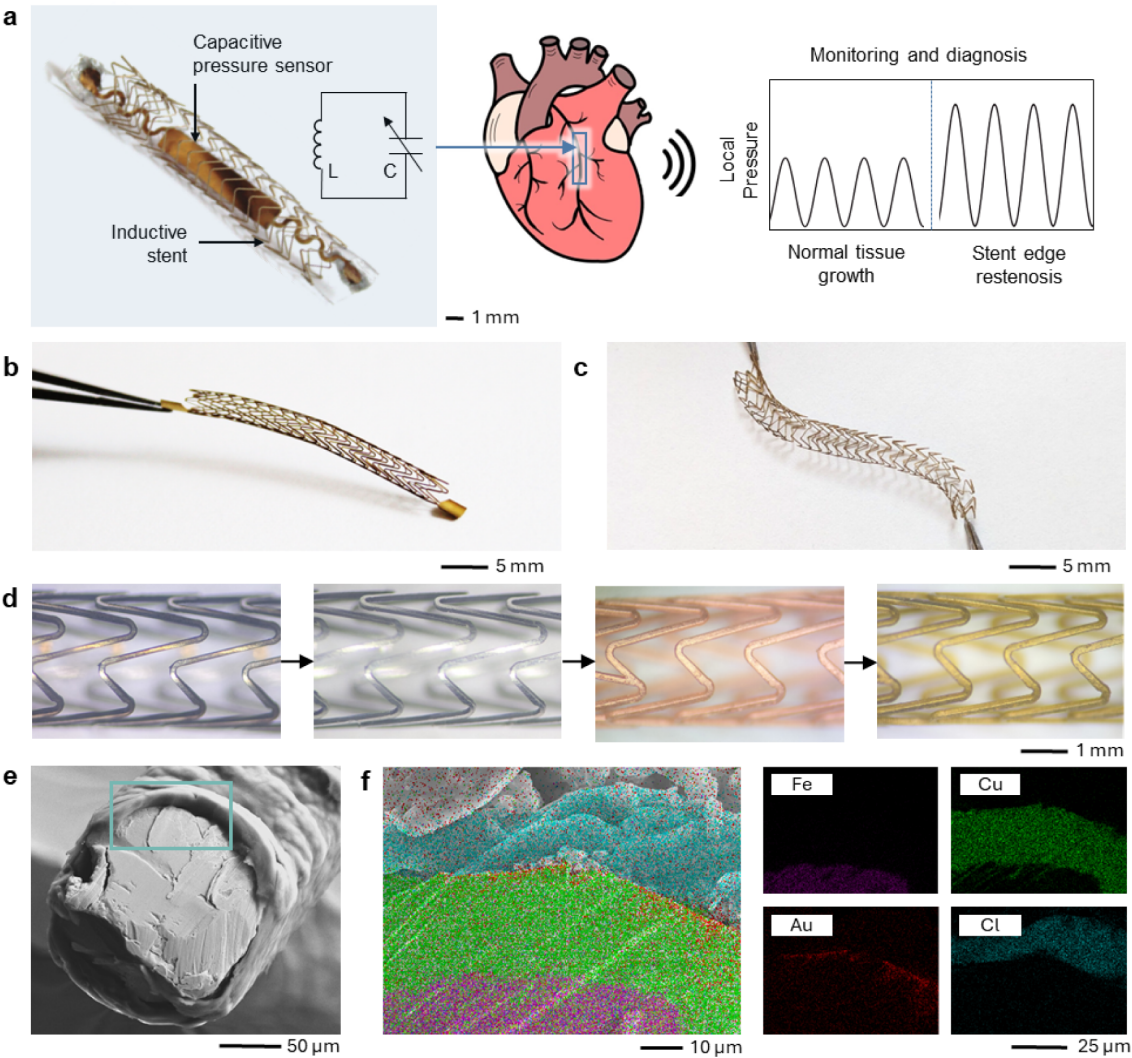
Material characterization and device fabrication. (a) Diagram of system overview showing stent and sensor forming a resonant circuit and sending a wireless signal to detect abnormal local pressures that would indicate stent edge restenosis. (b) Image of unexpanded inductive stent after postprocessing and (c) expanded stent after expansion with a 4 mm balloon catheter. (d) Images of stent struts throughout postprocessing, starting with the left image that shows the stent after laser cutting, then electropolishing, copper electroplating, and last, gold electroplating on the right. (e) FE-SEM cross-sectional image of a sliced, fully coated stent along with (f) EDS results showing distinct material layering of the stent with stainless steel (Fe), copper coating (Cu), gold coating (Au) and parylene coating (Cl).

### Device Optimization for Blood Pressure Detection

The capacitive pressure sensor can detect fluctuations in blood pressure for every heartbeat. It is built from copper foil with a thin layer of polyimide serving as an insulating layer between the plates (Figure 2a). A micropyramid dielectric is sandwiched between the two conductive plates to construct the pressure-sensing component of the stent. Serpentines are incorporated to provide axial stretchability, so the sensor can flex and stretch with the stent (Figure 2b). The sensor is designed with a micropyramid dielectric to enhance its sensitivity to shifts in pressure. This adjustment in dielectric structure is a proven way to increase pressure sensor sensitivity.^21–23^ This is especially important to diagnose SER, which has slight variances in local pressure as the tissue develops over time. The dielectric structure was evaluated with an optical profilometer. This fabrication demonstrates consistent pyramid sizes of roughly 120 μm in height and 150 μm wide (Figure 2c,d). This geometry prioritized for highest sensitivity of the capacitive sensor in the blood pressure region expected in the coronary arteries, optimizing the balance between sensitivity and reliability across various pressures.^24,25^ The sensor was then characterized under flow conditions to ensure that the capacitance could detect and respond to the shifts in pressure (Figure 2e). It also exhibited consistent results in capacitance through 1,000 cycles, with its baseline shifting from 1.35 to 1.45 pF after the cyclic testing, equivalent to 7% baseline capacitance shift (Figure 2f). While drift does exist for the sensor, the relative change in capacitance, not the absolute baseline value, is the key parameter influencing the frequency shift and overall sensor functionality. Since the sensing mechanism is based on relative signal changes, the observed baseline shift does not impair performance or reliability in practical scenarios.

**Figure 2:**
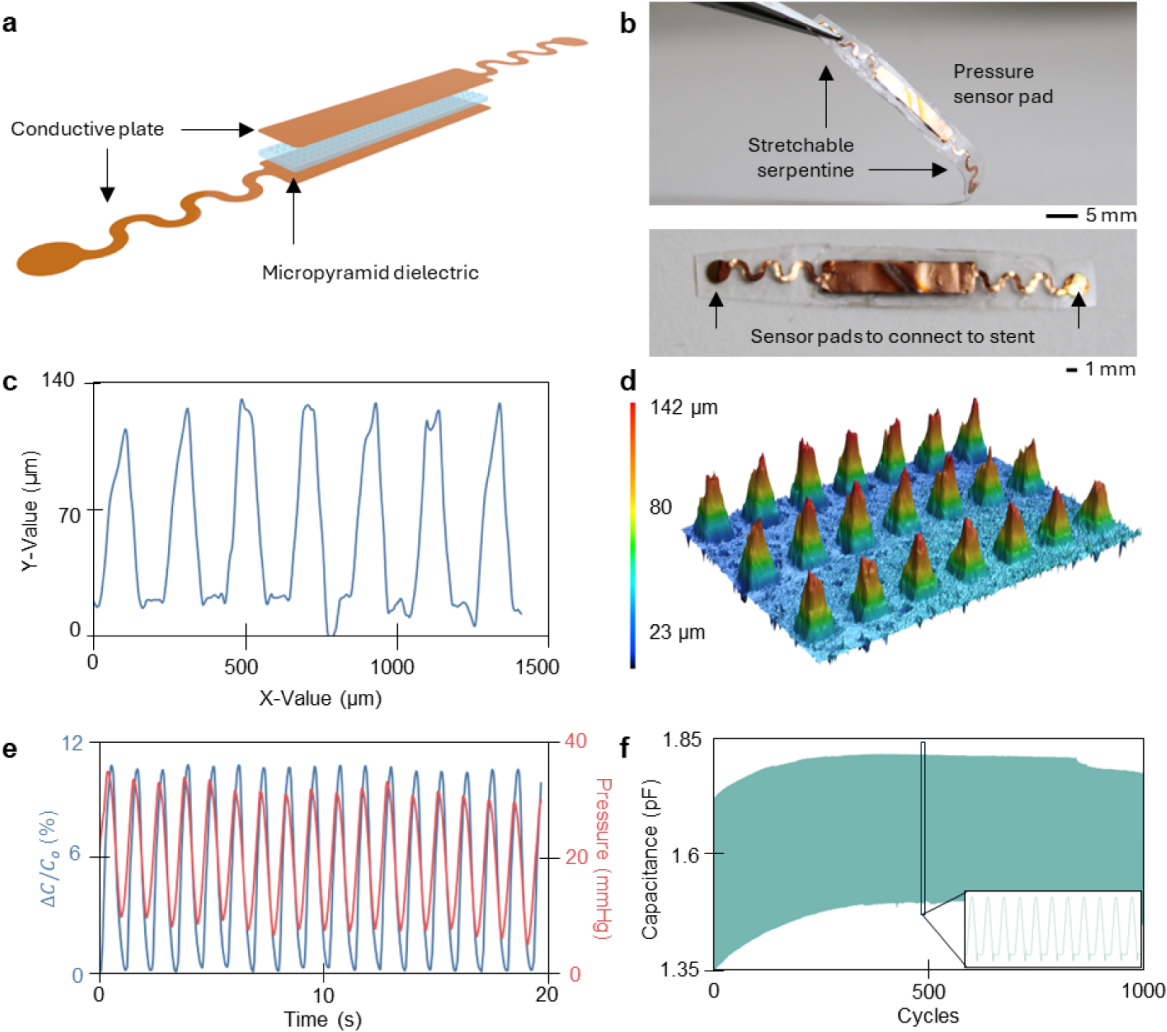
Sensor assembly and performance validation. (a) Exploded diagram of the capacitive sensor with the three distinct layers of two conductive plates (Cu) and a PDMS micropyramid dielectric sandwiched between them. (b) An image of the fully assembled sensor encapsulated in a thin layer of PDMS and showing where the sensor connects to the stent in the final assembly. (c) Height and widths of the micropyramid results taken with a profilometer along with a (d) 3D-profile visual. (e) A graph of the sensor capacitance fluctuating with varying pressure from a pulsatile flow machine to display correlation between the two parameters. (f) A plot of long-term cyclic of 1000 cycles to show reliability of the sensor overtime.

### System Integration and Simulation Studies

This work uses incident plane waves to communicate with the implanted device. The low-pitch-angle helix-like structure can be modeled as a meander dipole, and its radar cross-section (RCS) value is evaluated analytically.^26^ In our application, we have a limited degree of freedom in choosing the radius of the device. Additionally, the use of high-frequency electromagnetic waves is not desirable due to the lossy nature of human tissue. As a result, we designed an additional variable capacitive sensor connected to the stent, creating a resonant LC tank circuit. Such a self-resonant structure is a great candidate for reradiating electromagnetic waves in electrically small devices.^27^ The method to measure the wireless signal is considered low-power and is calculated to be below the exposure limits set by the IEEE.^28^ Additionally, the exposure duration to the EM waves is brief and noncontinuous. Therefore, the measurement theoretically poses a limited safety risk to biological tissue or implanted devices; however, it should be studied further.

After the sensor and stent have been individually assembled, the sensor is threaded through the stent and electrically connected at the ends with silver paint (Figure 3a-c). The ends are then encapsulated in PDMS to secure the sensor and ensure biocompatibility (Figure S7). The flexibility of the sensor allows it to be placed flush along the inner edge of the stent, ensuring that there is no flow obstruction during implantation. To measure the proposed system wirelessly, the experimental setup consists of a transmit/receive (Tx/Rx) antenna, the stent-sensor, a performance network vector analyzer (PNA), and a laptop (Figure 3d). The experiment is conducted within a semienclosed anechoic chamber to eliminate the potential reflections contaminating the data and to prevent electromagnetic wave exposure to the human body. The wave emitted by the antenna impinges on the device. The sensor, which is designed to be a self-resonant structure, induces current from the external electromagnetic wave at its resonance frequency and reradiates efficiently back to the Tx/Rx antenna. The backscattered signal is captured by the same antenna and PNA. The postprocessing consists of four steps: background (BG) subtraction, inverse Fourier transform (IFFT), time-gating, and fast Fourier transform (FFT). BG subtraction and time-gating are proven techniques for microwave-range measurements.^29,30^ BG subtraction requires recording of the BG data without the object of interest. The same process is then repeated to collect data when the object is present. We assume that the physical displacement of the cable and surroundings during the experiment is much smaller than the center wavelength (15 cm). Therefore, subtracting these two sets of data removes the cable reflection and cluttering effect coherently from the surroundings. Furthermore, time-gating is used after BG subtraction. After the time-domain spectrum of the entire measurement space is obtained, the location of the target is found relative to the antenna phase center. A filter window is then used to remove the reflections and further improve the signal quality. The orange curve shown in Figure 3e displays unprocessed data where two dominant peaks are found in the spectrum, as indicated by the arrows. The first corresponds to the discontinuity between the coaxial cable and the waveguide (WG) while the second corresponds to the discontinuity between the antenna and air. The blue curve shown in the figure is the data after background subtraction. A target response appears about 30 cm away from the antenna phase center. The reflections from the antenna and cable are reduced by over 50 dB. However, the reflection can still be seen in the time spectrum. Therefore, a time window (dashed window in the figure) is applied to the target signal to filter the reflection further and improve the signal-to-noise ratio. The filtered frequency spectrum in Figure 3f demonstrates that the device provides a peak backscattering wave at 2 GHz.

**Figure 3:**
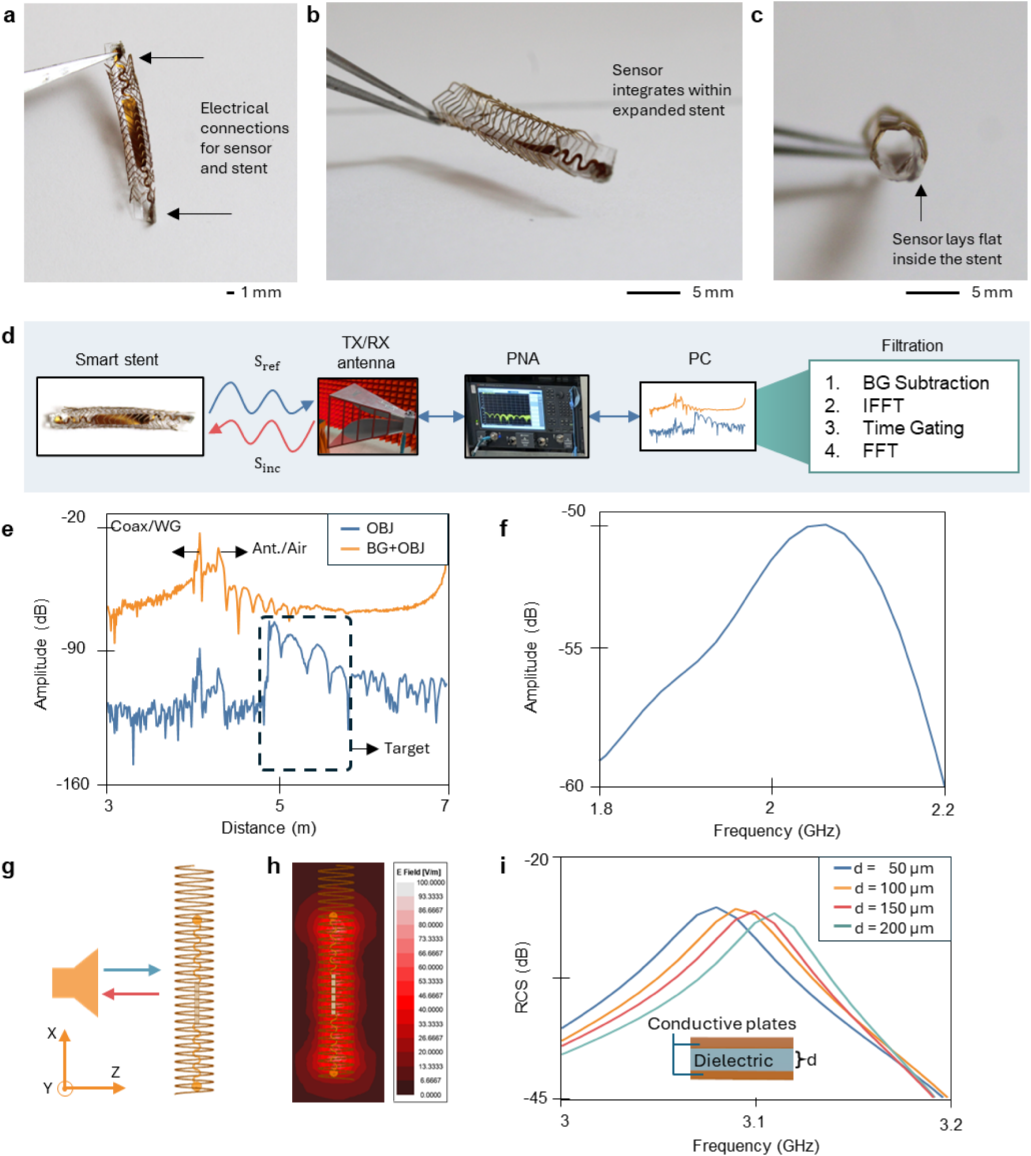
Experimentation setup with simulation studies. (a–c) Images of fully assembled stent and sensor, with sensor pads electrically connected to the stent to form the final LC circuit and the sensor laying flush within the stent. (d) Block diagram of wireless communication system that shows the antenna reading the reflections of the smart stent and postprocessing with a PNA and filtration through a PC. (e) Time domain data for raw data collected by the PNA (orange curve) and after background subtraction (blue curve). (f) Frequency domain data after gating displaying a clear resonant frequency of the stent. (g) Simulation setup in HFSS with applying incident plane waves directed at the simplified stent-sensor system. (h) Scattered electric field intensity plot at the resonance frequency (3.08 GHz) in the *x*–*z* plane. (i) Simulated RCS value for different dielectric thicknesses.

To validate the concept of using plane waves as an excitation method, a full-wave electromagnetic simulation is conducted in the ANSYS High Frequency Structure Simulator (HFSS). Using a simplified spiral model to represent the stent, an x-polarized plane wave impinges upon the device, and the scattered field is computed (Figure 3g). As shown in Figure 3h, a resonant mode is excited in the sensor region by the external plane wave. By decreasing the thickness, *d*, of the dielectric plate, the peak RCS value shifts down 10 MHz per 50 μm decrease (Figure 3i). This trend demonstrates the fundamental working principle of the device. Physiological changes cause shifts in the capacitance value, which then impact the resonant frequency that is being monitored. Furthermore, the effect of different incident angles in the azimuth plane (y-z) was stimulated and can be seen in Figure S8. Our work shows that the resonance frequency of the device remains unchanged with different incident angles. This suggests the robustness of the proposed reading method, as the axial rotation of the stent’s implantation would not impact the results. The main trade-off in postprocessing is the measurement time and the measurement quality, in terms of noise floor and time-domain resolution. Typically, the lower the intermediate frequency bandwidth (IFBW) of the PNA, the lower the noise floor. However, maintaining well-shaped low-frequency IF filtering requires a longer sweeping time. Meanwhile, the time per measurement cycle is proportional to the frequency number of points (fnpts), while the detection range in the time domain is also proportional to fnpts.^31,32^ Therefore, the PNA input parameters are chosen accordingly for the different measurements.

### Measurement of Wireless Distance Shifts and Catheter Pressurization

Most wireless data was assessed at a 30 cm distance between the stent-sensor system and the receiving antenna (Figures 4a,b and S9). The receiving antenna was incrementally moved in 10 cm steps to characterize the system’s device limitations until the stent’s signal was no longer detectable (Figure 4c,d). The signal is lost at 60 cm, which shows the system can successfully be detected at 50 cm away, farther than other papers have previously reported. This can be seen in Table 1, which displays a summary of battery-free wireless antennas for physiological monitoring that have been reported in literature.^7–13,33–35^ This work stands out as this new reading method overcomes the distance limitations commonly associated with inductive coupling in wireless setups. This new method provides an increased signaling range of over 50 cm through air while maintaining a quality factor of 8.9. To evaluate this method further, a balloon catheter was used to pressurize the capacitive sensor inside the stent, while keeping the rest of the system unchanged. The balloon was threaded through the device and incrementally inflated to provide higher pressure for the stent to experience (Figure S10). As shown in Figure 4e, the resonant frequency curve shifts to a lower frequency with increased balloon pressure. This aligns with what is expected; as capacitance increases with an increase in pressure, the frequency should decrease in value. Additional measurements can be seen in Figures S11-S12, taken from one stent at multiple levels of balloon catheter inflation. This trend also matches the simulation results discussed in the previous section. To ensure that the signal displayed is coming from the stent, Figure 4f displays four variations performed with the different components of the test. A catheter was positioned in front of the antenna and measured in both its deflated and inflated states to confirm the absence of any signal within the same frequency range. The only resonant frequencies on this plot come from when the stent is in front of the receiving antenna, as indicated by the arrows.

**Figure 4:**
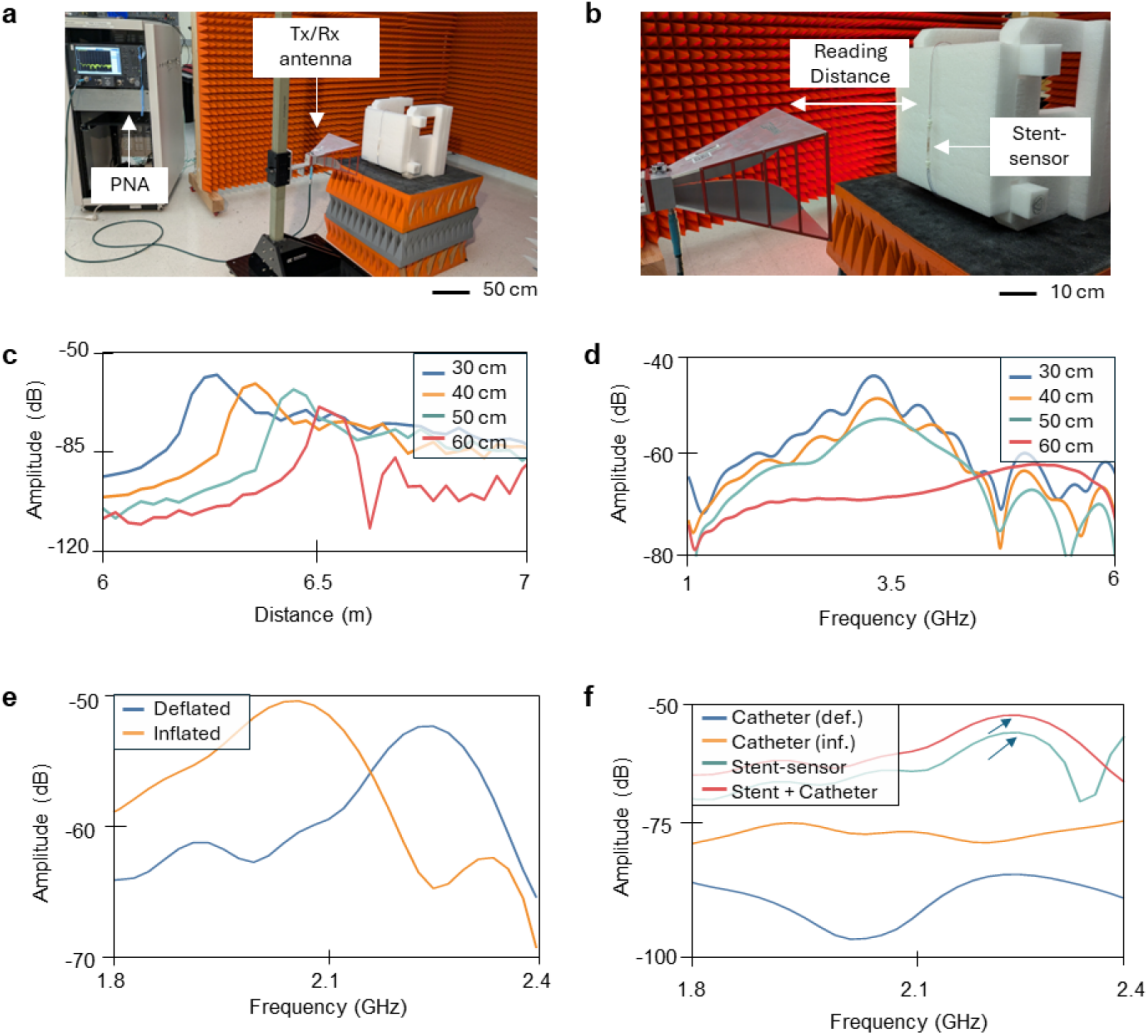
Physical experimental setup and balloon catheter testing. (a,b) Images of measurement setup displaying the Tx/Rx antenna placed 30 cm away from the stent and connected through air, surrounded by noise absorbers. (c) Plot showing the amplitude of reflections as distance is varied and (d) frequency results showing resonant frequency up to 50 cm away as antenna is moved incrementally from 30 to 60 cm away from the stent-sensor system. (d) Frequency results from balloon catheter testing of the deflated and inflated balloon and (e) results that highlight the shift of resonant frequency when the stent is placed in front of the antenna compared to when the stent is absent but other equipment remains. (f) Additional frequency results displaying the peak in frequency shift when the stent is being measured, while no frequency peak exists without the transmission of the stent.

**Table 1:** Comparison of Battery-Free Wireless Antennas for Physiological Monitoring

Reference	Signaling range	Field type	Communication method	Device type	Frequency shift with Δ100 mmHg
This work	> 50 cm air	Far-field	Monostatic backscatter	Integrated stent platform	10 MHz
Cleven et al.^8^	10 cm air	Near-field	Inductive coupling	Additional sensor implant	-
Tang et al.^35^	10 cm tissue	Near-field	Ultrasonic sensing	Additional drug injection	0.57 MHz
Herbert et al.^18^	2.5 cm air	Near-field	Inductive coupling	Integrated stent platform	11 MHz
Chen et al.^34^	2.5 cm water	Near-field	Inductive coupling	Additional sensor implant	15 MHz
Boutry et al.^33^	-	Near-field	Inductive coupling	Additional sensor implant	0.66 MHz
Son et al.^13^	1 cm air	Far-field	Backscatter	Integrated stent platform	-
Park et al.^11^	0.8 cm air	Near-field	Inductive coupling	Integrated stent platform	12.5 MHz
Oyunbaatar et al.^10^	20 mm air	Near-field	Inductive coupling	Integrated stent platform	0.1 MHz
Rigo et al.^12^	-	Near-field	Inductive coupling	Integrated stent platform	25 MHz
Chen et al.^7^	-	Near-field	Inductive coupling	Integrated stent platform	0.41 MHz

### In Vitro Measurement of SER Diagnosis

After the pressure shifts were successfully detected, the system was evaluated for physiological signal monitoring using a pulsatile flow pump to simulate blood flow (Figures 5a,b and S13). The stent was encapsulated in Ecoflex tubing to mimic arterial implantation (Figure S14). Normal testing was performed with the no-stenosis model, so no obstructions appeared in the flow line. The stenosis testing introduced an obstruction that modeled 50% SER after the stent-sensor. The presence of this blockage downstream of the sensor affects the local pressure change for every heartbeat. 50% of the flow will be blocked from passing through the restenosis, so a buildup of pressure occurs at the sensor location in the stent, which allows for wireless detection of tissue buildup by monitoring the relative change in signal.

**Figure 5:**
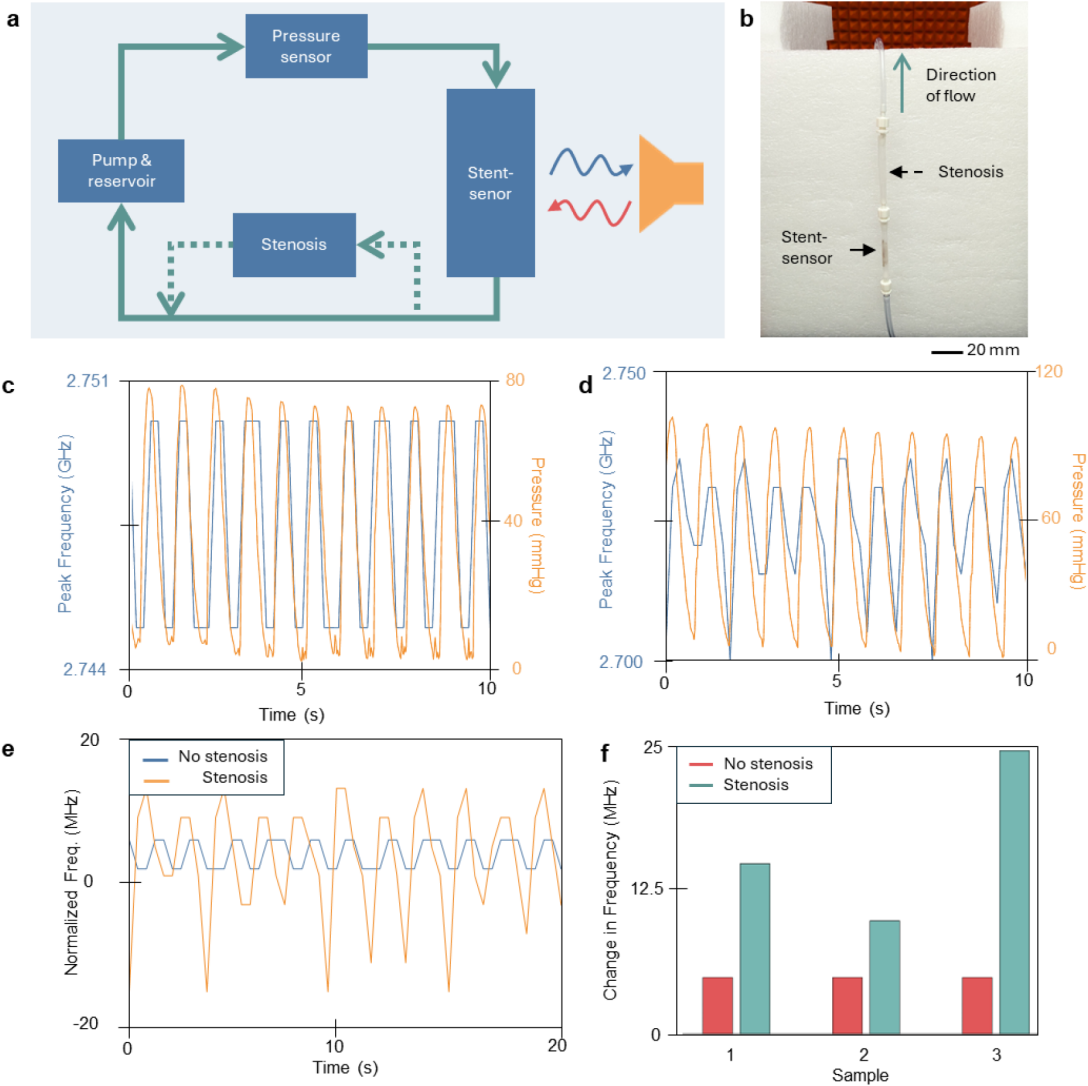
In vitro flow testing and SER detection. (a) Block diagram of the pulsatile flow setup and (b) image of flow testing when stenosis is placed directly after the stent-sensor to mimic stent-edge restenosis. (c) Wireless frequency data from the stent-sensor system and corresponding pressure data from the commercial sensor when stenosis is not present in the system and (d) same data when stenosis is placed directly after the stent. (e) A plot of the direct wireless frequency comparison shows the magnitude difference between normal flow and stent-edge restenosis. (f) A bar plot that shows different samples (*n* = 3) to display repeatability of results.

A standard pulsatile flow test with DI water was performed first. As the pressure increases in the system, the frequency shifts to a lower frequency value. The stents were seen to shift roughly 5 MHz for a 50–60 mmHg pressure shift (Figure 5c), while staying consistent through the cycling period. After the standard flow testing, a stenotic mold was added directly after the stent to occlude the outflow vessel by 50% in diameter. This increased the local pressure seen in the sensor, shifting the overall resonant frequency. As shown in Figure 5d, this frequency shift was roughly 2–3 times larger than that observed during the standard flow testing. This result is summarized in Figure 5e,f, where multiple stents were assessed and showed consistent results in diagnosing poststent stenosis.

## Conclusions

This paper reports on a set of materials and methods for the far-field wireless detection of SER using an implantable bioelectronic stent. By utilization of optimized biocompatible materials, the system features a fully flexible and highly sensitive capacitive pressure sensor that integrates seamlessly with the mechanical and geometric constraints of vascular stents. Beyond stent applications, this sensor concept can be scaled for broader use, although challenges such as maintaining sensitivity over larger areas, mechanical robustness, and signal stability must be addressed. Scalable fabrication techniques and further dielectric optimization may offer promising pathways to enable larger-scale applications. The implementation of radar backscattering allows for a monostatic readout approach, significantly extending the wireless interrogation range to over 50 cm without the need for precise antenna alignment. Computational simulations and in vitro experiments affirm the effectiveness of the antenna-stent architecture as a platform for localized pressure monitoring. Further studies will optimize the sensor for potential earlier restenosis detection. The system should also be studied for long-term stability testing, including phantom models and FEA studies to run fatigue analysis and optimize the design of the system to ensure safe implantation. Additionally, we will evaluate the system in different media to refine the measurement technique to better mimic in vivo conditions before it is finally assessed in vivo. This method facilitates real-time tracking of hemodynamic changes and advances clinical translation by addressing critical challenges associated with wireless implant performance. By combining advanced materials processing with functional sensor-stent interfaces, this system paves the way for scalable, continuous, and noninvasive diagnostics for high-risk cardiovascular patients.

## Experimental Setup

### Fabrication of Inductive Stents

The stents were made from a stainless-steel tube (304SS 14XX; Vita Needle Inc.) and cut using a femtosecond laser cutter (OPTEC) with a rotational stage. The laser was set to 65% power of 4 W with 5–6 cut repetitions and a speed of 3.65 mm/s. Afterward, the stents were cleaned by sonication in a distilled (DI) water bath for 1 min. They were then electropolished using stainless steel electropolish solution (E972; ESMA) for 1 min at a current of 0.5 A. After the stents were sonicated again in DI water, they were electroplated with copper and gold to lower their electrical resistance. To prepare for electroplating, a copper wire was threaded through the stent lumen to promote a uniform deposition. The stent was first electrocleaned in an acidic electrocleaning solution for 15 s at 5 V. A surface activator was then used at 6 V for 30 s to hydrate the surface and improve the wettability. Copper plating was performed first to enhance gold adhesion, using a copper sulfate (CuSO_4_)-based electrolyte at 1.4 V until the stent resistance dropped below 7 Ω, typically requiring about 3 min. Finally, gold was electroplated on top of the copper at 2.7 V for 1 min using a gold chloride (HAuCl_4_)-based electrolyte. After plating, the stent was expanded with a 4 mm balloon catheter.

### Assembly of Capacitive Sensors with a Stent

The sensors were assembled with copper film (MSE PRO, 6 μm thick), polyimide ink (PI; PI2545), and polydimethylsiloxane (PDMS; Sylgard 184, Dow Corning). PDMS was spin-coated onto a glass slide at 1500 rpm for 30 s and cured on a hot plate at 100 °C for 20 min. The slide was then laminated with copper film, ensuring the removal of air bubbles to achieve a flat and uniform surface. It was subsequently plasma treated to create a hydrophilic surface for the PI. The PI was spin-coated at 1250 rpm for 1 min and then cured at 240 °C on a hot plate for 1 h. The film was then cut using a lasercutter (OPTEC) to define 2 mm × 10 mm sensor areas with serpentines to stretch the remaining length of 27 mm, allowing it to fit inside the stent. The patterned electrode was peeled off and transferred onto a separately cured PDMS slide. A microstructured dielectric layer was placed on top of the sensor portion of the electrode, followed by another electrode to form a parallel-plate capacitive pressure sensor. A final PDMS layer was placed over the sensing portion, leaving the two electrode ends exposed, which were electrically connected to the stent. The sensor was sealed with uncured PDMS and then cured on a hot plate at 100 °C for 20 min. It was then cut off the slide and threaded through the expanded stent. The stent ends were connected to the sensor ends using quick-drying silver paint (Fast Drying Silver Paint; Ted Pella), then sealed with uncured PDMS and cured with a heat gun at 180 °C. The dielectric layer was a microstructured pattern to enhance the sensitivity to pressure. To make this pattern, an acrylic mold was patterned with a femtosecond laser. Uncured PDMS was spin-coated onto the acrylic mold at 1000 rpm for 1 min and then cured in a 65 °C oven for 4 h.

### Simulation

The domain of interest was set to a 75 × 75 × 75 mm air box to construct the simulation. A linearly polarized incident plane wave was excited from the top surface in the *z*-direction with polarization aligned along the *x*-axis, matching the stent sensor orientation. The stent was modeled as a copper helix in HFSS, with a wire radius of 73 μm, a helix radius of 2.3 mm, a pitch of 980 μm, and 18.25 turns. PI was used as the dielectric plate material (*ε*_*r*_ = 4.6, *tanδ* = 0.004). The frequency sweep ranged from 1 to 4 GHz with 301 points.

### Wireless Measurement

The balloon catheter testing allows for enough time to achieve high time-domain resolution, a long detection range, and a low noise floor. As a result, the measurement parameters were set to a frequency range of 1–18 GHz with 801 fnpts and an IFBW of 200 Hz, resulting in approximately 10 s per measurement. For the flow-based measurement, a narrower bandwidth of roughly 0.5 GHz, fnpts of 51, and IFBW of 1 kHz were chosen to reduce the measurement time to 0.2 s per measurement.

### Antenna Distance Measurement

To evaluate wireless signal sensitivity at various distances, the antenna (RF Sprin DRH18) was moved incrementally farther away from the device by 10 cm at a time. Background data (without the stent) were recorded at each step for postprocessing calibration. Distance measurements were conducted from the antenna’s phase center to the stent sensor.

### Balloon Catheter Testing

For a proof-of-concept test, the stent-sensor system was assessed using a balloon catheter setup. A 4 mm balloon catheter was threaded through the stent and then gradually inflated to various pressures to induce a change in capacitance. Frequency shifts were recorded using the same wireless antenna configuration, with pressure adjustments made manually via the balloon catheter pump.

### Flow Testing

The expanded stent-sensor system was implanted in a silicone (Ecoflex 30, Smooth-On) artery mold with an inner diameter of 3.8 mm and a wall thickness of 2 mm, which dimensions previously shown to mimic simplified elastic, linear coronary artery biomechanics.^36^ This system used comparable size tubing to the pulsatile flow machine (Series 1400, Harvard Apparatus) to give roughly 40 mmHg pressure change at 60 BPM using DI water (stroke volume ∼ 1.5 mL). To monitor pressure during the flow process, a commercial pressure sensor (Honeywell 26PCBFB6G) was used, with voltage output captured using a digital multimeter (Model 2100, Keithley) and tracked in real time via LabVIEW. Wired testing was completed by encapsulating an assembled pressure sensor in the arterial mold and monitoring fluctuations in capacitance with an LCR meter (B and K 891). Wireless frequency shifts were recorded with the stent positioned 30 cm from the antenna. To simulate stenosis, a mold with 50% occlusion was 3D-printed (Elegoo Saturn 2) and integrated into the silicone artery downstream of the stent. The mold was filled with Ecoflex-30 and cured for 2 h in a 65 °C oven before testing under flow conditions.

## Supplementary Material



## Notes

Additional fabrication images of system, electroplating resistance results, EDS studies, simulation studies, images of testing setup, wireless results, in vitro test images (MP4)
